# An in vitro fermentation model to study the impact of bacteriophages targeting Shiga toxin-encoding *Escherichia coli* on the colonic microbiota

**DOI:** 10.1038/s41522-022-00334-8

**Published:** 2022-09-26

**Authors:** Graça Pinto, Sudarshan A. Shetty, Erwin G. Zoetendal, Raquel F. S. Gonçalves, Ana C. Pinheiro, Carina Almeida, Joana Azeredo, Hauke Smidt

**Affiliations:** 1 CEB - Centre of Biological Engineering, University of Minho, 4710-057 Braga, Portugal; 2 LABBELS - Associate Laboratory, Braga/Guimarães, Portugal; 3 Laboratory of Microbiology, Wageningen University & Research, Stippeneng 4, 6708 WE Wageningen, The Netherlands; 4 INIAV, IP-National Institute for Agrarian and Veterinary Research, Rua dos Lagidos, Lugar da Madalena, Vairão, Vila do Conde Portugal; 5 LEPABE - Laboratory for Process Engineering, Environment, Biotechnology and Energy, Faculty of Engineering, University of Porto, Rua Dr. Roberto Frias, 4200-465 Porto, Portugal; 6 Present Address: Department of Medical Microbiology and Infection Prevention, University Medical Centre Groningen (UMCG), Groningen, The Netherlands

**Keywords:** Metagenomics, Applied microbiology, Microbiota, Next-generation sequencing, Antimicrobials

## Abstract

Lytic bacteriophages are considered safe for human consumption as biocontrol agents against foodborne pathogens, in particular in ready-to-eat foodstuffs. Phages could, however, evolve to infect different hosts when passing through the gastrointestinal tract (GIT). This underlines the importance of understanding the impact of phages towards colonic microbiota, particularly towards bacterial families usually found in the colon such as the *Enterobacteriaceae*. Here we propose in vitro batch fermentation as model for initial safety screening of lytic phages targeting Shiga toxin-producing *Escherichia coli* (STEC). As inoculum we used faecal material of three healthy donors. To assess phage safety, we monitored fermentation parameters, including short chain fatty acid production and gas production/intake by colonic microbiota. We performed shotgun metagenomic analysis to evaluate the outcome of phage interference with colonic microbiota composition and functional potential. During the 24 h incubation, concentrations of phage and its host were also evaluated. We found the phage used in this study, named *E. coli* phage vB_EcoS_Ace (Ace), to be safe towards human colonic microbiota, independently of the donors’ faecal content used. This suggests that individuality of donor faecal microbiota did not interfere with phage effect on the fermentations. However, the model revealed that the attenuated STEC strain used as phage host perturbed the faecal microbiota as based on metagenomic analysis, with potential differences in metabolic output. We conclude that the in vitro batch fermentation model used in this study is a reliable safety screening for lytic phages intended to be used as biocontrol agents.

## Introduction

Shiga toxin-producing *Escherichia coli* (STEC) is a potentially deadly pathogen found in food and impacts millions of people worldwide. As estimated by the World Health Organization (WHO), STEC caused more than one million cases of illness, more than 100 deaths and about 13,000 Disability Adjusted Life Years (DALY) in 2010^1,2^. Despite the improvements in food safety, with the implementation of Good Manufacturing Practices (GMP) and Hazard Analysis Critical Control Point (HACCP), the number of foodborne illness is still significant^3^. Bacteriophages, or phages for short, use bacteria as their host for their multiplication and, because of their lytic activity, they have been considered for the biocontrol of foodborne pathogens^4^. Several lytic phages targeting STEC strains have been isolated and characterized for their lytic activity^5–8^. Lytic phages are also compatible with the One health approach, which suggests the integration of human medicine, veterinary medicine and environmental science^9^. Phages could be used for food decontamination, for pathogen control in the environment, but also to treat foodborne infections in humans. In the context of STEC infections, because antibiotics are not recommended for therapy^10,11^, phage biocontrol is seen as a vital alternative. STEC strains are characterized by the presence of Shiga toxin gene, which is acquired by *E. coli* through the insertion of a prophage, known as Shiga phage. Toxin production is linked to the induction and excision of Shiga phages. Some antibiotics are known to induce Shiga phages, which can increase the risk of developing haemolytic-uremic syndrome (HUS) disease^10^. On the other hand, lytic phages were already shown to be able to control the growth of STEC strains without inducing this response^7,12^.

Lytic phages are considered safe for human microbiota, due to their high specificity usually targeting a single species^13^ or closely related species (e.g. *E. coli* O157:H7 and *Salmonella enterica*^14^). Nevertheless, in a recent study^15^, it was reported that a virulent phage infecting *E. coli*, was able to perform host jumps during passage through the gastrointestinal tract (GIT) in a mouse model. The phage needed to adapt to an intermediate host since the original host had a fitness decrease^15^. This adaptation demonstrates the importance to fully understand and characterize the safety of lytic phages targeting species of the *Enterobacteriaceae* family, since this family includes common members of the human GIT microbiota^16^. Over the last years, the importance of the GIT microbiome for human health became evident, as the microbiome has been linked to digestion, protection against pathogen colonization, but also, to mental diseases and other conditions^17,18^. Therefore, it is important to guarantee that no adverse disturbances within the human microbiota are triggered by the use of phages.

Finding a reliable in vitro model that would allow a first screening of phage safety towards the GIT microbiota is crucial, especially because human and animal model studies, besides having ethical issues, are laborious and expensive^19^. Moreover, animal models pose some limitations, since the GIT microbiota is different from that of humans, and therefore may not reflect the real impact of phages^19^. Even though in vitro fermentation models lack host factors, they provide some advantages, as it is possible to perform dynamic sampling over time and they are reproducible^20^. As the inoculum, faecal microbiota is used in most cases, and incubation is done in vessels where the anoxic atmosphere is controlled^21^.

In this study, we propose an in vitro model that uses faecal slurry from healthy donors to characterize the impact of phage preparation on the colonic microbiota. We used a previously isolated and characterized phage, named phage vB_EcoS_Ace (Ace)^12^, which is highly specific towards serotype O157:H7, the most known STEC serotype. Furthermore, this phage was shown to be able to infect other closely related species, strains of *Citrobacter freundii* and *Shigella sonnei*^12^. Phage Ace belongs to the *Demerecviridae* family, more specifically to the *Tequintavirus* genus. Even though phage Ace presents a long latency period of 55 min, and a low burst size of only 19 PFU per infected cell, it was showed to have a good lytic activity against the planktonic cells of its host, which makes it a good candidate to be used for biocontrol^12^. The proposed model was used with the faecal content of three healthy donors. Experiments were further monitored by measuring the production of short chain fatty acids (SCFA), important metabolites produced by the colonic microbiota^22^, and gases being produced and consumed, including hydrogen and carbon dioxide. The concentration of phage Ace and its host was also monitored during fermentation. Finally, to fully understand the impact on the microbiome, shotgun metagenomic analysis was performed.

## Results

### Phage titre decreases during simulated in vitro digestion

**Fig. 1:**
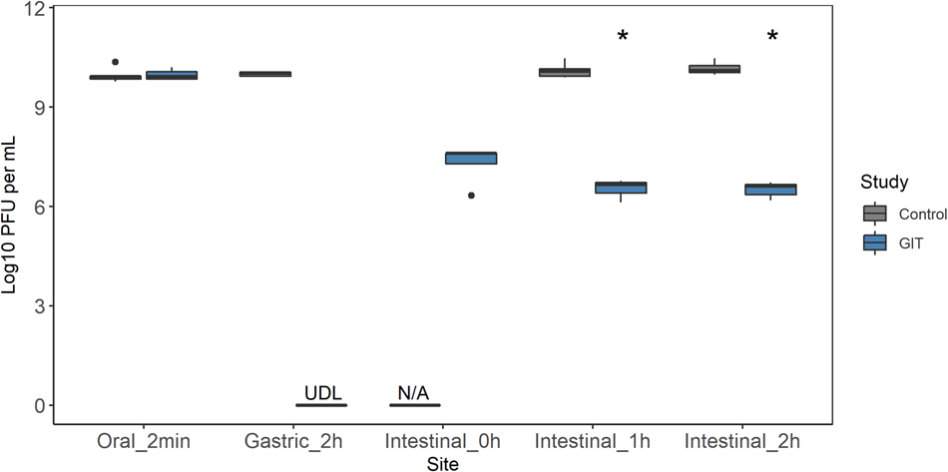
Phage behaviour during simulated gastrointestinal tract passage. The passage of phage Ace was simulated using the standardized static in vitro digestion method. The concentration of phage was measured by the end of each phase: oral, gastric, and intestinal. During the intestinal phase the concentration was also measured at the beginning and after one hour of incubation. Boxplots show the median, the upper and lower quartiles and the highest and lowest values; three independent experiments performed in duplicate are represented in each boxplot. Statistical differences comparing the concentration of phage in control and during GIT simulated transit (*ρ < 0.001) were assessed using *t* test of rstatix pipeline of R using the Two-samples unpaired test option.

A standardized static in vitro digestion method^23^ was used to simulate phage Ace passage through the upper GIT (Fig. 1). Phage Ace was added to the system at a high concentration (about 10 log PFU/mL). Curiously, at the end of the gastric phase it was not possible to quantify the phage. However, when the transition for the intestinal phase was achieved, phage Ace was quantified to be about 6.5 log PFU/mL. The concentration remained stable for the duration of this phase (1 and 2 h post intestinal phase initiation).

To better understand the inactivity phenomenon at the gastric phase, phage Ace particle size and Zeta values were measured under control (phage suspension SM buffer) and gastric phase conditions at pH 3 and 7 (Table 1). The size of phage Ace particles change considerably (ρ < 0.001) at gastric pH 3 phase (115.5 ± 2.786 nm in control vs. 8250 ± 1675 nm at pH 3). Furthermore, the size distribution was also different, switching from homogenous to a heterogenous suspension, as indicated by the polydispersity index (PdI) values, which were 0.1 for control and 0.7 at gastric pH 3. When increasing the pH value in the gastric phase to 7, the size of phage particles decreased to values close to control conditions albeit still different (ρ < 0.001; 210.7 ± 48.87), however, the distribution remained heterogeneous with a PdI of 0.4. The Zeta values of phage Ace were different for the distinct conditions tested, changing from −13.3 under control conditions to −4.45 and −4.61 under gastric conditions, independently of pH value (ρ < 0.001).

**Table 1:** Size, polydispersity index (PDI) and the zeta (Ζ) values of phage Ace at control (SM Buffer) and Gastric conditions.

Size (nm)	PDI	*Ζ*
**Control**		
115.5 ± 2.786	0.1 ± 0.006	−13.3 ± 0.411
**Gastric pH3**		
8250 ± 1675^a^	0.7 ± 0.2^a^	−4.45 ± 0.775^a^
**Gastric pH7**		
210.7 ± 48.87^a^	0.4 ± 0.09^a^	−4.61 ± 1.22^a^

^a^Means are significantly different (ρ < 0.001) compared to control value.

### Phage titre increases only in the presence of its host

**Fig. 2:**
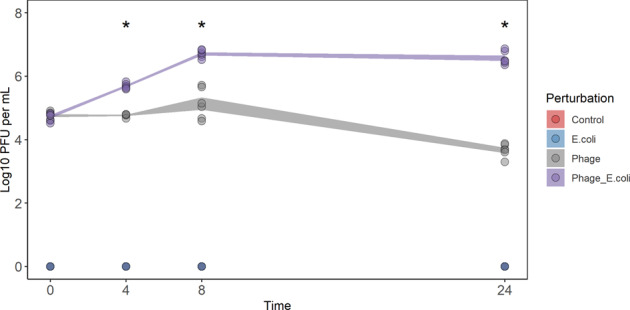
Dynamics of phage Ace during in vitro batch fermentation. The concentration of phage Ace (Log_10_ PFU/mL) during in vitro batch fermentation was measured for 0, 4, 8 and 24 h of incubation. The data of all three experiments were combined in this plot, and standard deviation is shown. Statistical differences comparing phage concentration for perturbations only phage (Phage) or phage in combination with host (Phage_E.coli) (*ρ < 0.05) were assessed using *t* test of rstatix pipeline of R using the Two-samples unpaired test option.

**Fig. 3:**
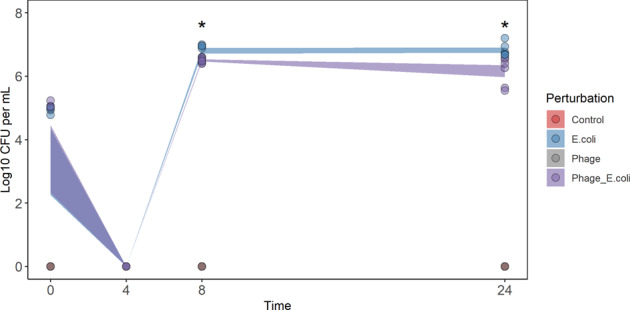
Dynamics of *E. coli* 5947 during in vitro batch fermentation. The concentration of *E. coli* 5947 (Log_10_ CFU/mL) during in vitro batch fermentation was measured for 0, 4, 8 and 24 h of incubation. The data of all three experiments were combined in this plot, and standard deviation is shown. Statistical differences comparing *E. coli* 5947 concentration for perturbations only *E. coli* (E.coli) or phage in combination with host (Phage_E.coli) (*ρ < 0.05) were assessed using *t* test of rstatix pipeline of R using the Two-samples unpaired test option.

To mimic colonic conditions, SIEM medium (described to be similar to proximal colon content^24^), and anoxic conditions were used. Phage Ace was quantified during fermentation, at the following time points: 0, 4, 8 and 24 h (Fig. 2). Phage concentration only increased when the host (*E. coli* 5947) was also added to the serum bottles (Phage_E.coli). In fact, phage Ace in the absence of its host (Phage) lost its infection capability by the end of the fermentation. At 8 h of fermentation, a slight increase in phage titre was observed, however, this was not observed at the end of fermentation. Persistence of *E. coli* 5947 during fermentation was also assessed (Fig. 3). Remarkably, the concentration at the initial time point (0 h) for the incubation with faecal microbiota of donor D1, for the first experiment, and at time point 4 h of all experiments performed, the CFU count of *E. coli* 5947 was below detection limit (100 CFU). For the other time points, i.e. 8 and 24 h, *E. coli* 5947 had increased both in the presence and absence of phage Ace, albeit slightly less (about 0.5 log CFU/mL) when in the presence of phage (ρ < 0.05). For both perturbations (phage Ace and *E. coli* 5947 addition), the individuality of donor faecal microbiota did not interfere with the outcome, as seen by the low standard deviation.

The abundance of phage Ace genes was assessed mapping its genome against the shotgun metagenomic reads. DNA sequences of phage Ace were only detected when phage was added to the system. Moreover, in the presence of the host, there was a 10-fold increase in abundance, indicating that phage Ace replication occurred only when *E. coli* 5947 was present. This was valid for all three individual experiments (Supplementary Fig. 1).

### Phage did not change taxonomic microbiota composition

**Fig. 4:**
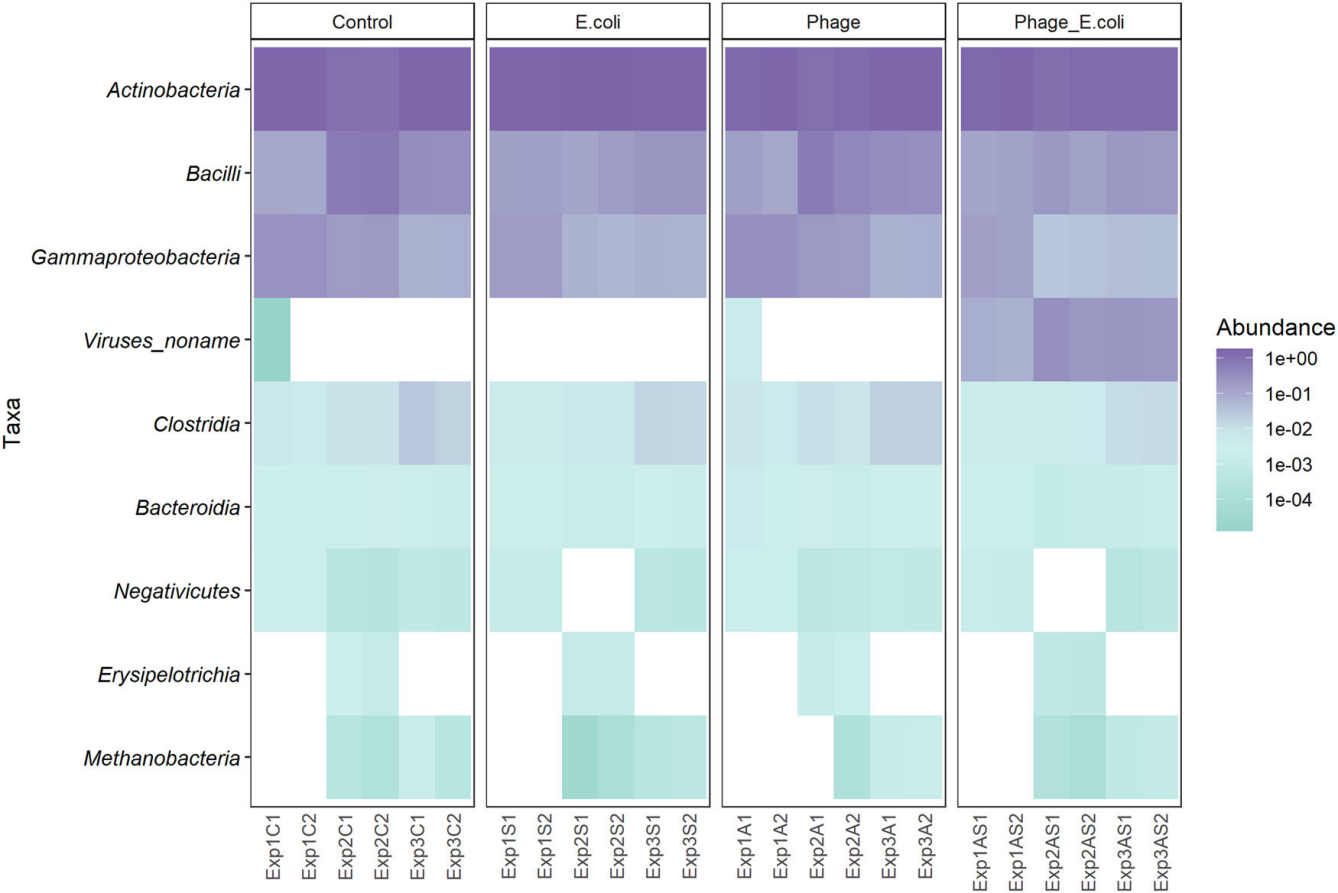
Relative abundance at Class rank level by the end of fermentation. The resulting microbiota for all perturbations and donors’ fermentations is represented in a heatmap with the relative abundances.

**Fig. 5:**
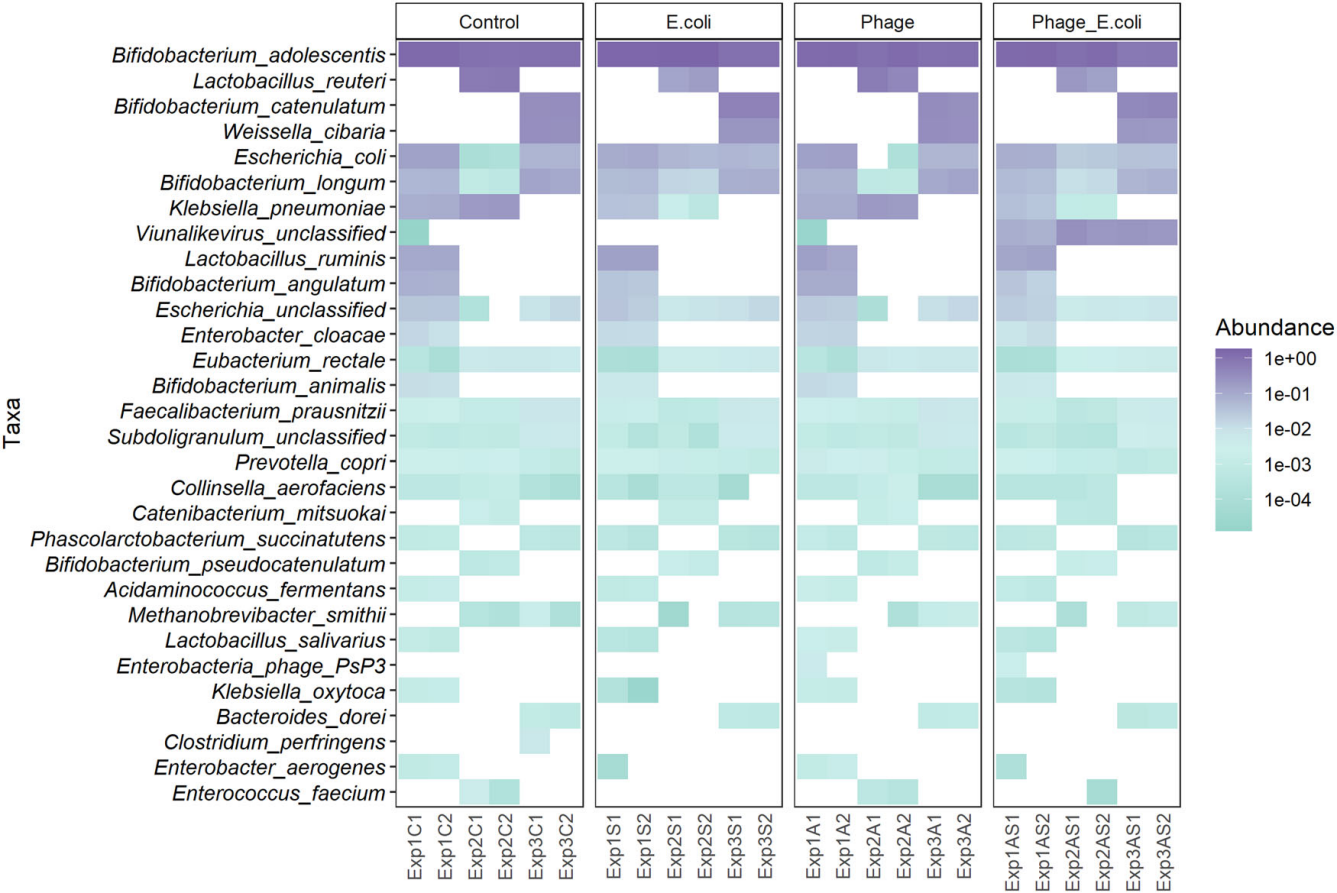
Relative abundance at Species rank level by the end of fermentation. The resulting microbiota for all perturbations and donors’ fermentations is represented in a heatmap with the relative abundances.

Shotgun metagenomic analysis showed that the colonic microbiota did not change in composition with the addition of phage Ace; however, the addition of *E. coli* 5947 altered the colonic microbiota. The taxonomic ranks Class and Species were used to construct abundance heatmaps, as shown in Figs. 4 and 5, respectively. These heatmaps, particularly the Species heatmap, displayed the individuality of the three donors’ microbiota used in this study. All three inocula were composed predominantly of *Actinobacteria*, divided into *Bifidobacterium adolescentis* and *B. longum* (for all donors’ microbiota), *B. catenulatum* (D3 microbiota), and *B. angulatum* (D1). This was followed by *Bacilli*, which included the species *Lactobacillus reuteri* (D2) and *Weissella cibaria* (D3). The third Class most represented was the *Gammaproteobacteria*, mostly *E. coli* (in all donors’ microbiota), *Klebsiella pneumoniae* (D1 and D2) and *Escherichia* unclassified (all donors). At the taxonomic rank of Class, it was also possible to detect Viruses, which were found at a higher abundance when phage Ace and *E. coli* 5947 were added together, as compared to when only phage Ace was added.

**Fig. 6:**
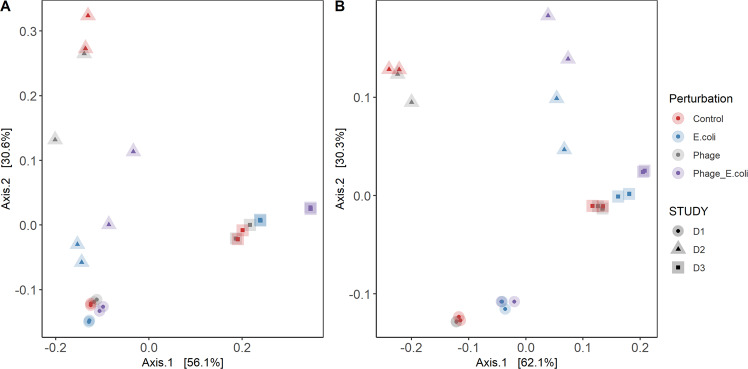
Beta diversity analysis of microbiota composition and pathways after perturbations. Principal coordinate analysis (PCoA) plots of beta diversity for (**A**) composition and (**B**) pathways abundances. The plots exhibit the clustering to understand microbial taxonomic and functional (pathways) composition based on pairwise Bray–Curtis dissimilarities of each perturbation and donors’ faecal microbiota.

The addition of *E. coli* 5947 (alone or simultaneously with phage Ace) did not have a significant effect on the reduction of observed taxa (*ρ* > 0.05; Supplementary Fig. 2). The inequality of samples (dominance Gini index) was significantly affected by the addition of *E. coli* 5947 (*ρ* < 0.05). When phage Ace was added simultaneously, there were no changes observed in the inequality of samples compared to the control condition. The dominant genera for all conditions were *Bifidobacterium* and *Lactobacillus*, independently of the conditions tested. On average, six taxa contributed to 95% of the abundance for control and phage conditions, and only 4.5 taxa when *E. coli* 5947 was added (both in the absence and in combination with phage Ace). Beta diversity was visualized by ordination plot (PCoA), indicating that the individuality was maintained independently of the perturbations introduced to the systems (Fig. 6A), with a *ρ* < 0.01. Moreover, the faecal microbiota composition of D2 was the most affected by the addition of *E. coli* 5947.

**Fig. 7:**
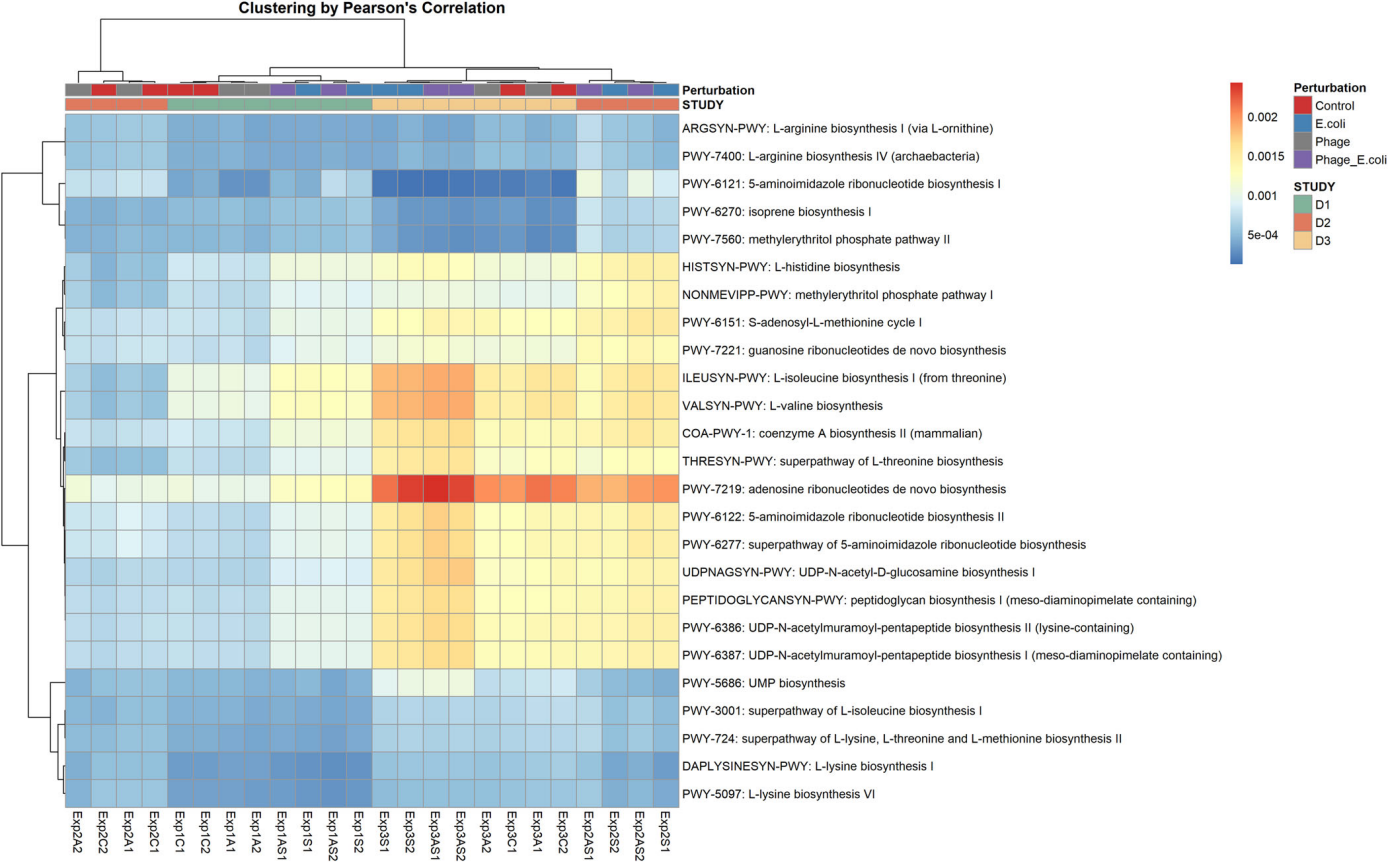
Relative abundance of top-25 pathways by the end of fermentation. Pathways were detected using the HUMaN2 pipeline. Clustering of pathways, and of each experiment is represented in the plot.

The HUMaN2 pipeline was used to decipher, which pathways were detected at the end of fermentation. This analysis also demonstrated a clear interference rendered by the presence of *E. coli* 5947. The top 25 pathways detected were represented in a heatmap and clustered by Pearson’s correlation (Fig. 7). The addition of *E. coli* 5947 led to changes in the pathways’ abundances, which did not occur by the addition of phage Ace. Beta diversity of pathways (Fig. 6B) revealed that individuality was maintained during the experiment (with a *ρ* < 0.01), and the pathways detected for D2 were the most affected by *E. coli* 5947. Taxonomic composition (Fig. 6A) and the pathways (Fig. 6B) detected correlated, as determined using the Mantel statistic based on Pearson’s product-moment correlation with Pearson’s r of 0.816 and a *ρ*-value of 0.001. As the only source of carbohydrates added to the medium was starch, the starch degradation V (PWY-6737) metabolic pathway was analysed in more detail (Supplementary Fig. 3). For D1 and D3, *E. coli* taxa were the biggest contributor to this pathway, reducing when *E. coli* 5947 was added. For D2, *E. coli* taxa were not a contributor to this pathway.

## Discussion

Phages used for food safety, particularly when applied in ready-to-eat food, will be ingested by the consumer, and inevitably will go through the GIT. Therefore, in this study, we used an in vitro batch fermentation model to elucidate the impact that a STEC infecting phage has on the colonic microbiota of healthy donors. Previous studies have used this in vitro system to study for example the effect of prebiotics^25–27^ or antibiotics^28^ on colonic microbiota.

Phage titres and proliferation can be significantly reduced by low pH values^29^, which could mean a reduction of phage concentration during GIT transit, particularly due to stomach acidity^30^. Nevertheless, there are phages, as the one used in this study^12^, that are able to tolerate low pH values^31,32^. Moreover, other studies have reported that some foodstuffs can function as protective allies for phage to passage the stomach^30^, therefore, the likelihood of having viable phage particles reaching the intestine is very high. To further investigate phage Ace behaviour during passage through the GIT, a model that mimics upper gastrointestinal digestion^23^ was used to understand the amount of phage that would reach the lower intestine. A highly concentrated phage Ace suspension (10 log PFU/mL) was introduced into the GIT simulation system. By the end of the simulation, about six log PFU/mL of phage suspension reached the intestine, staying stable until the end of this phase (2 h of incubation). Remarkably, it was not possible to count any phage particles during the gastric phase, but as phage was active in the intestinal phase, this phenomenon was further investigated. We observed that acidic gastric conditions neutralized the charge of phage, which induced phage aggregation leading to a drastic decrease in the phage titre. We propose that the aggregation observed is the reason why phage Ace is protected from the acidic condition found in stomach.

These findings imply that phage Ace, and others with similar aggregation behaviour, when ingested with food, will eventually reach the intestine. It is known that the intestine, particularly the large intestine, harbours a complex microbiota that among other functions plays a major role in digestion^33^ and is one of the protective barriers against pathogen colonization^34^. In this study, we used an in vitro fermentation model to screen the safety of the STEC specific phage Ace towards colonic microbiota fermentation. The phage host, the bacterium *E. coli* 5947, was also used as perturbation. As proof of concept, the faecal content of three healthy donors was used as inoculum to understand the role of colonic microbiota individuality on the outcome of phage Ace safety.

The dynamics of phage Ace and *E. coli* 5947 during fermentation was monitored. Phage Ace persisted in the system, in the presence and absence of its host; however, it only increased its concentration when the host was also added. The addition of phage Ace did not change faecal microbiota composition however, the presence of *E. coli* 5947, alone or in combination with phage, had a profound effect on the faecal microbiota composition. This effect was particularly evident when the faecal content of donor two (D2) was used. This indicates that the individuality of faecal microbiota indeed has a role on the perturbation outcome. The foreign *E. coli* strain added had a higher impact when indigenous *E. coli* abundance was lower, as was the case for D2. Although a transcriptomic or proteomic analysis was not performed that would be required to describe the activity of specific pathways, we were able to monitor the gas composition during fermentation (Supplementary Figs 4 and 5) and qualitatively assess potential differences in abundance of pathways during fermentation based on metagenomic data using HUMaN2^35^. To this end, our qualitative analysis seems to indicate that the fermentation metabolite production was not affected by the presence of phage Ace, which is aligned with the stability of the microbiota composition in the presence of the phage. However, and interestingly, the addition of an external *E. coli* to the faecal samples seems to impact fermentation as an increase of acetate production was observed, particularly in the faecal samples from D2 and D3 (Supplementary Fig. 5). Moreover, the contribution of *E. coli* to the starch degradation pathway in the presence of *E. coli* CECT 5947 appears to be reduced, which was not observed in the overall *E. coli* population abundances. This probably indicates that a competition between the foreign and the indigenous *E. coli* strains occurred. This competition could have led to the differences observed in relative abundances of members of the colonic microbiota and resulted in different fermentations. Moreover, in contrast to the bacteria present in the faeces, *E. coli* 5947 was cultivated in laboratory media before inoculation and, thereby, could have had a competitive advantage. Although we cannot completely rule out this advantage, we assume this it is not very likely since *E. coli* was grown in LB medium under oxic conditions which largely deviate from growth conditions in SIEM medium.

When looking into the β diversity of all samples, it was noticed that individuality was retained, even when *E. coli* 5947 was added, even though we observed that for D2 there was a shift in microbiota composition when *E. coli* 5947 was added. Metagenomic sequence analysis revealed that the different inocula used in this study were different, yet, all were predominated by *Bifidobacterium* and *Lactobacillus* genera, which are not targets for phage Ace infection^12^. Although SIEM medium is considered a good simulation of adult chyme entering the colon, a study demonstrated that the medium could lead to an increase of some *Bifidobacterium* spp^19^. Therefore, other media should be used in future studies to confirm the results obtained in this study.

While analysing the shotgun metagenomics data, it was possible to assess the abundance of phage Ace genome, which had a more pronounced increase when added simultaneously with its host. This, once again, indicated that phage Ace did replicate when the host was present, demonstrating that in the three faecal inocula used, phage Ace did not find a suitable host for replication. Nevertheless, De Sordi et al. reported in 2017, that the microbiota was a possible intermediate for host jump of phage P10. As the host has less advantage in a complex community such as the gut microbiome, phage P10 had evolved to infect another host during GIT passage. This host jump was not accompanied by a reduced ability to infect the original host^15^.

The complexity of microbiota, obtained from the faecal content of healthy donors, is a good inoculum to study the behaviour of phages^21,36^. This type of inoculation allows the testing of a vast microbiota composition, in a simple manner. It is known that the study of a complex community as a whole gives more information than when pure cultures are used, regarding the role of each component to the ecosystem^37^. The use of three faecal inocula from different donors elucidated the importance of testing the phage safety for different microbiota, and, with a wider sampling (extending the number of donors), the results could be easily extrapolated. However, to improve this model, different media should be used to guarantee that bias is not introduced during the in vitro fermentation. The results obtained in this study are in line with other reports^38^, which demonstrated the safety of *E. coli* specific phages when applied in a simulated human small intestinal model system, inoculated with a consortium of seven bacterial species.

Hsu et al.^39^ reported that in a gnotobiotic mouse model, phages were able to modulate a bacterial consortium (composed by selected human gut commensal bacteria). By interfering with the abundance of its host, phages were able to change the overall concentration of certain bacteria, which resulted in a direct consequence for the gut metabolome^39^. In contrast to our model, the mouse model allows the monitoring of changes in the microbiome/ metabolome in a longitudinal manner. It is important to notice that each phage will behave differently, especially if a complex microbiota is used, increasing the probabilities of encountering a suitable host. So, even though phage Ace did not lead to differences in the three microbiota used in the in vitro model proposed, this does not imply that another phage will have a similar outcome. The next step for phages that are shown to be promising in the proposed in vitro model, i.e. without leading to perturbations in a large set of different microbiota tested, is the study of its behaviour in a longitudinal in vitro model. The model herein presented, should be seen as a first step for safety analysis, and a more complex in vitro model, that allows for longitudinal studies, should be pursued. Nevertheless, it is important to guarantee the usage of complex microbiota, as the one used in this study.

In this study, we have demonstrated that a phage infecting STEC O157 serotype has no impact on the healthy colonic microbiota and therefore, together with previously reported complementary evidence of safety^12^, can be considered a safe biocontrol agent. Furthermore, the in vitro model used in this study provided an easy, non-expensive and reliable way to evaluate the impact of phages on the human gut microbiota. The impact of a perturbation could be assessed through the monitoring of fermentation parameters. So, we propose that this model should be considered as a standard safety assay to evaluate phages intended to be used for foodborne pathogen biocontrol.

## Methods

### Bacterial strain and culture conditions

*E. coli* phage vB_EcoS_Ace (Ace), previously isolated and characterized^12^, and strain *E. coli* O157:H7 CECT 5947 (Δ*stx2::cat*) were used in this study. Both phage Ace and *E. coli* 5947 were propagated at 37 °C in LB broth (all reagents are from Sigma-Aldrich (Merk), Darmstadt, Germany), with or without agar (Merck, Darmstadt, Germany) (1.2% for the bottom layer and 0.4% for soft agar overlays). Chloramphenicol (Sigma) supplementation at 25 µg/mL, was used as selective marker for *E. coli* CECT 5947 enumeration in LB agar plates during the in vitro fermentations.

### Static in vitro digestion model

Phage Ace passage through the upper GIT was simulated using a standardized static in vitro digestion method described before^23^. Briefly, 5 mL of phage Ace suspension (500 µl of a suspension containing approximately 1 × 10^10^ PFU/mL was diluted in 4.5 mL of Saline-Magnesium (SM) Buffer (5.8 g/L NaCl, 2 g/L MgSO_4_.7H_2_O, 50 mL/L 1 M Tris pH 7.5)) was mixed with Simulated Salivary Fluid (SSF) at a final ratio of 1:1 (v/v). In this study, α-amylase was excluded since starch was lacking in the samples. The oral phase had a duration of 2 min. The gastric phase was accomplished by adding Simulated Gastric Fluid (SGF) at a ratio of 1:1 (v/v) to the previous mix, porcine pepsin (Sigma-Aldrich) (final concentration 2000 U/mL), and CaCl_2_ (to achieve 0.075 mM final concentration). HCl (1 M) was added to the mixture until a pH of 3.0 was reached. Ultra-pure water was added to reach the correct dilution of SGF. The gastric phase had a duration of 2 h. The intestinal phase was initiated by adding Simulated Intestinal Fluid (SIF) in a ratio of 1:1 (v/v) to the previous mixture. The mixture was neutralized (pH 7.0) by adding 1.0 M NaOH. Pancreatin solution (Sigma-Aldrich) (800 U/mL), fresh bile (Sigma-Aldrich) (160 mM) and CaCl_2_ (0.3 M) were added to the mixture. Again, ultra-pure water was used to achieve the correct dilution of SIF. This last phase had a duration of 2 h. All phases were carried out at 37 °C under an agitation at 120 rpm, in a water bath with an integrated shaker (Certomat^®^ WR, Sartorius, Gottingen, Germany). Samples of 500 µL were taken after each phase, and phage viability was assessed by phage plaque forming unit (PFU) counts by the microdrop method^40^, with some modifications. Briefly, a lawn of the host (*E. coli* CECT 5947) was prepared using 100 µL of an overnight cell culture in 3 mL of soft agar and allowed to dry. After, 10 µL of 10-fold serially diluted phage suspensions were placed onto the lawn and allowed to run down.

Note that all reagents, simulated fluids, and sample were pre-heated to 37 °C in a water bath. The composition of the three simulated fluids^23^ is further described in detail in Supplementary Methods.

Phage Ace size distribution and zeta potential (charge) were measured by dynamic light scattering (DLS) using a Malvern Zetaziser, Model NANO ZS (Malvern Instruments Limiter, Worcestershire, UK). Size was analysed at 25 °C in a polystyrene cell, and zeta potential was analysed in a folded capillary cell^29,41^.

### Study design and sampling

The growth medium used in this study was the Standard Ileal Efflux Medium (SIEM, Tritium Microbiology, Veldhoven, The Netherlands)^42^. SIEM composition was modified by removing all carbohydrates with the exception of soluble starch from potato (Sigma-Aldrich), as it is known that it is highly abundant in the large intestine^43^. SIEM medium was prepared with the following composition per 1000 mL: 50 mL BCO medium; 16 mL Salts; 10 mL MgSO_4_.7H_2_O; 7.4 g Starch; 0.5 g Cysteine; 823 mL Distilled water; 100 mL MES 1 M; 1 mL Vitamin solution and 1 mL Resazurin. BCO medium and Vitamin solution were purchased at Tritium Microbiology.

The faecal samples used in this study were collected from three healthy donors living in Wageningen (the Netherlands) and surrounding area, used in three independent experiments denominated as D1, D2 and D3. Samples were collected directly into a closed box with an anaerobic strip, and transferred to an anaerobic chamber to guarantee microbiota integrity^44^. Twenty five percent of faeces were prepared using 7 g of sterile Dial (Tritium Microbiology), 35.7 g distilled water, 9.8 sterile glycerol and 17.5 g of faeces, and maintained at −80 °C until used. Faecal samples were thawed and diluted in SIEM medium to achieve 1% (v/v) of faecal sample to be used as start inoculum for the in vitro fermentation. The samples were provided from another study for which no ethical approval was necessary. Written informed consent was obtained for all participants.

**Fig. 8:**
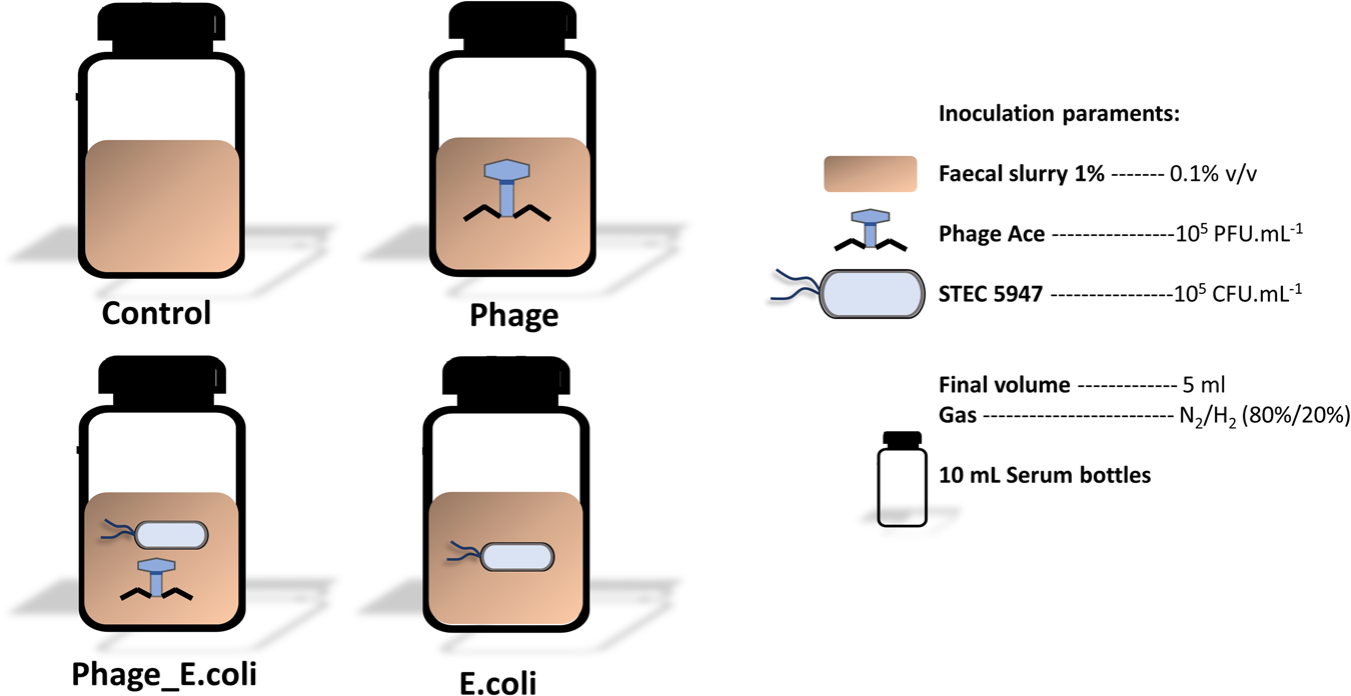
Overview of experimental design. Each serum bottle, in duplicate, was inoculated as depicted. Phage Ace and *E. coli* 5947 were added into the respective serum bottles, with the final concentration 10^5^ PFU/mL or CFU/mL, to obtain a multiplicity of infection (MOI) of 1. A control condition (no perturbation added) was included during the experiments. This was performed separately for each donor’s faecal content, named D1, D2 and D3, each representing one of the three donors.

In vitro fermentations were performed in duplicate^45^, with some modifications. Briefly, serum bottles of 10 ml were used, and the inoculation was performed using 4.5 mL of SIEM, 0.5 mL faecal slurry 1.0%, 0.5 mL of phage Ace at 10^7^ PFU/mL, and 0.5 mL of *E. coli* CECT 5947 at 10^7^ CFU/mL. Phage and *E. coli* were added to certain bottles as represented in Fig. 8. When needed demineralized water was used to complete the volume up to 5.0 mL. The different conditions tested were performed in duplicate in each experiment. The inoculation was performed in an anaerobic chamber (AS-580, Anaerobe Systems, California, USA) with a controlled atmosphere composed of 96% N_2_ and 4% H_2_, and the bottles were closed using rubber stoppers and secured with a metal crimp cap. The bottles were incubated in an incubator shaker (Innova 40, New Brunswick Scientific, Nijmegen, The Netherlands) at 37 °C, 100 rpm, during 24 h. Samples for bacterial and phage counts, gas chromatography (GC), high performance liquid chromatography (HPLC), and DNA extraction were retrieved at the beginning and after 4, 8 and 24 h of incubation. Sampling and analysis for each parameter are described in the following sections.

#### Phage and Bacteria quantification

For all time points, viable phage Ace and culturable *E. coli* CECT 5947 were quantified using the microdrop method. PFU counts were performed as described in section two. Colony forming unit (CFU) counts were performed to enumerate *E. coli* CECT 5947^40^. To avoid over-estimation of phage lytic activity, the samples were diluted 10-fold in a solution of 7.5 % w/v loose-leaf Black tea (330 µL) mixed with freshly prepared 5.0 mM Ammonium Iron (II) Sulfate hexahydrate (700 µL), and incubated at least for 5 min at RT^12^. This suspension was further 10-fold serially diluted, and 10 µL of each dilution was deposited onto an LB plus chloramphenicol agar plate and allowed to run down. All plates were incubated at 37 °C for 16 to 18 h.

#### Fermentation metabolite analysis

The cumulative gas production was analysed by GC^46^. Briefly, 100 µL of gas sample were taken using a 1.0 mL syringe and injected into a Compact GC 4.0 (Global Analyser Solutions, Breda, The Netherlands). The analysis was performed using the Chromeleon™ Chromatography Data System (CDS) Software (Thermo Scientific, Massachusetts, USA).

SCFA (propionate, acetate, and butyrate) and some intermediate organic acids (lactate, succinate) were analysed by HPLC using a Shimadzu LC2030C-Plus with a column KC811 (Shimadzu Corporation, Kyoto, Japan) at 45 °C. Standards used for calibration included an organic acids mix (composed of citrate, malate, succinate, fumarate), glycerol, and succinate. For calibration, the following standards were used: butyrate, propionate, succinate, lactate, and acetate. To prepare the samples, 1 mL aliquots of the in vitro fermentation at each time point were immediately frozen at −20 °C. At the time of HPLC analyses, samples were thawed and centrifuged at 14,000 × *g* for 5 min. The supernatant was carefully collected to avoid retrieval of particles. Two-hundred microliters of either samples or standards were mix with 200 µl of dimethyl sulfoxide (DMSO) in 0.01 N sulfuric acid (H_2_SO_4_), as the internal standard, by vortexing. The mix was then centrifuged (10,000 × *g*, 5 min), and 200 µl of each supernatant was analysed by HPLC in a 96-well plate. The eluent used in the HPLC was 0.01 N H_2_SO_4_, and the refractive index (RI) was used for detection. The results of this part of the study are presented in supplementary Figs. 4 and 5.

#### DNA extraction and sequencing

One mL of the in vitro fermentation at each time point was used for DNA extraction, being immediately centrifuged (11,000 × *g*, 10 min, 4 °C), and the pellet frozen (−20 °C) until used. Pellets were thawed on ice for approximately 30 min and dissolved in 700 µL of Stool Transport and Recovery Buffer (STAR, Roche Diagnostics Corporation, Indianapolis, USA). The suspensions were transferred to sterilized 2 mL screwed cap tubes containing one scoop of 0.1 mm zirconia beads and one scoop of 2.5 mm glass beads, and treated in a bead beater (room temperature, 5.5 m/s for 3 × 1 min) (Precellys 24, Bertin Technologies, Montigny-le-Bretonneux, France). The suspensions were heated for 15 min at 95 °C under agitation (100 rpm), subsequently centrifuged (14,000 × *g* for 5 min at 4 °C), and the supernatants were kept. Another cycle of bead beating was performed, adding 300 µL of STAR buffer to the same screw cap tubes used before. The supernatants of both cycles were combined, and 250 µL were used for genomic DNA extraction using a Maxwell 16 Tissue LEV Total RNA Purification Kit Cartridge (Promega, Wisconsin, EUA). The DNA concentration was determined using Qubit™ dsDNA BR assay (ThermoFisher Scientific), following the manufacturer’s instructions. DNA samples with concentrations of about 30 ng/µL were sent for library preparation and shotgun metagenomic sequencing (Illumina PE150) at NovoGene Europe services (Cambridge, United Kingdom).

#### Genomic data analysis

Read sequences of the shot gun metagenomics were first trimmed using BBMap^47^ for removal of adapters, artifacts and PhiX read contamination. The quality of sequences was accessed through FastQC^48^. The trimmed sequences were analysed using the Metaphlan 2.0^35^ under HUMAnN 2.0^35^ pipeline for taxonomic analysis, and HUMAnN 2.0 pipeline for functional analysis.

The data generated by Metaphlan was analysed in R Studio version 1.3.959 (https://www.rstudio.com/) with R version 4.0.1 (https://cran.r-project.org/), using the R packages Microbiome^49^ and Vegan^50^. Using the Microbiome package, alpha diversity indexes, dominance index gini and observed taxa, were determined for each experiment (donor). Statistical analysis comparing all diversity parameters with each other was performed by Wilcox test from the Microbiome package. The Vegan package was used to generate a Constrained Analysis of Principal Coordinates based on pairwise Bray–Curtis dissimilarities. The statistical analysis of this data was performed using Anova within the Vegan package. PERMANOVA significance test within Vegan package was used to calculate group-level differences (Beta diversity). Vegan was also used to calculate alpha diversity Shannon-Weaver index of functional data obtained from HUMAnN 2.0 analysis. Moreover, plots were obtained for ratios of different pathways for each donor and condition (perturbation).

To check for phage Ace genome presence in the trimmed sequence reads^51^, the mapping of the latter was performed using the tool HTSeq^52^.

### Statistical analysis

All statistical analysis was performed in R version 4.0.1 embedded in R Studio version 1.3.959, using the pipeline rstatix: Pipe-Friendly Framework for Basic Statistical Tests version 0.7.0 (https://CRAN.R-project.org/package=rstatix). The *t* test was performed using the Two-samples unpaired test option, comparing two groups.

## Supplementary information

Supplementary Material

## Data Availability

The authors confirm that the relevant data are available within the paper and its Supplementary file. The sequencing data are available at the European Nucleotide Archive with accession number PRJEB49704.

## Code availability

Analysis codes and processed datasets are publicly available the GitHub repository; https://github.com/GracaP88/Pinto_et_al_Phage-gut.
