# Supplementary material for: An in vitro fermentation model to study the impact of bacteriophages targeting Shiga toxin-encoding Escherichia coli on the colonic microbiota
Source: NPJ Biofilms Microbiomes. 2022 Sep 26;8:74. doi: 10.1038/s41522-022-00334-8 (PMC9512901; doi:10.1038/s41522-022-00334-8)
Supplement: Supplementary file 1 — Supplementary Material [file 41522_2022_334_MOESM1_ESM.pdf]

An *in vitro* fermentation model to study the impact of bacteriophages targeting Shiga toxin-encoding *Escherichia coli* on the colonic microbiota.

Graça Pinto[1,2,3], Sudarshan Shetty[3$], Erwin G. Zoetendal[3], Raquel F.S. Gonçalves[1,2], Ana C. Pinheiro[1,2], Carina Almeida[1,2,4,5], Joana Azeredo[1,2*], Hauke Smidt[3*]

[1] CEB - Centre of Biological Engineering, University of Minho, 4710-057, Braga, Portugal.

[2]LABBELS - Associate Laboratory, Braga/Guimarães, Portugal

[3] Laboratory of Microbiology, Wageningen University & Research, Stippeneng 4, 6708 WE, Wageningen, The Netherlands.

[4] INIAV, IP-National Institute for Agrarian and Veterinary Research, Rua dos Lagidos, Lugar da Madalena, Vairão, Vila do Conde, Portugal

[5] LEPABE - Laboratory for Process Engineering, Environment, Biotechnology and Energy, Faculty of Engineering, University of Porto, Rua Dr. Roberto Frias, 4200-465 Porto, Portugal

* Corresponding authors:

Centre of Biological Engineering, Campus de Gualtar, 4710-057 Braga, Portugal. Tel: (+351) 253 604 424; e-mail: jazeredo@deb.uminho.pt

Laboratory of Microbiology, Wageningen University & Research, Stippeneng 4, 6708 WE, Wageningen, The Netherlands; e-mail: hauke.smidt@wur.nl

[$]Present Address: Department of Medical Microbiology and Infection Prevention, University Medical Centre Groningen (UMCG), Groningen, The Netherlands

Supplementary Figures

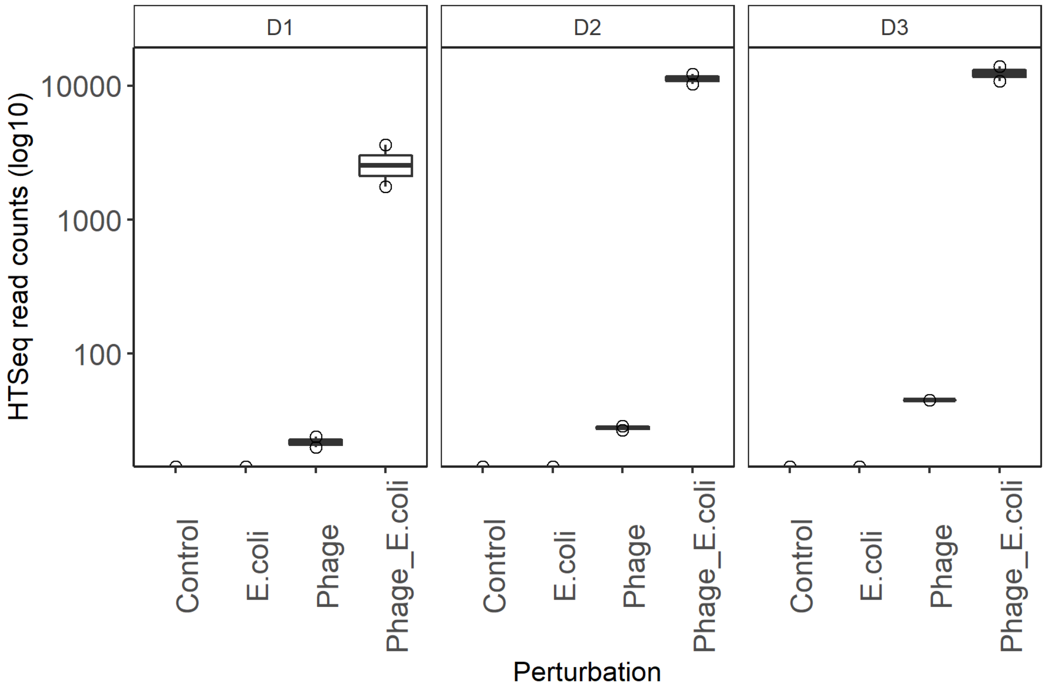

Supplementary Figure 1. Phage Ace sequence read abundances. The count of Phage Ace genes was performed for each experiment, using the pipeline HTSeq with the shot gun reads.

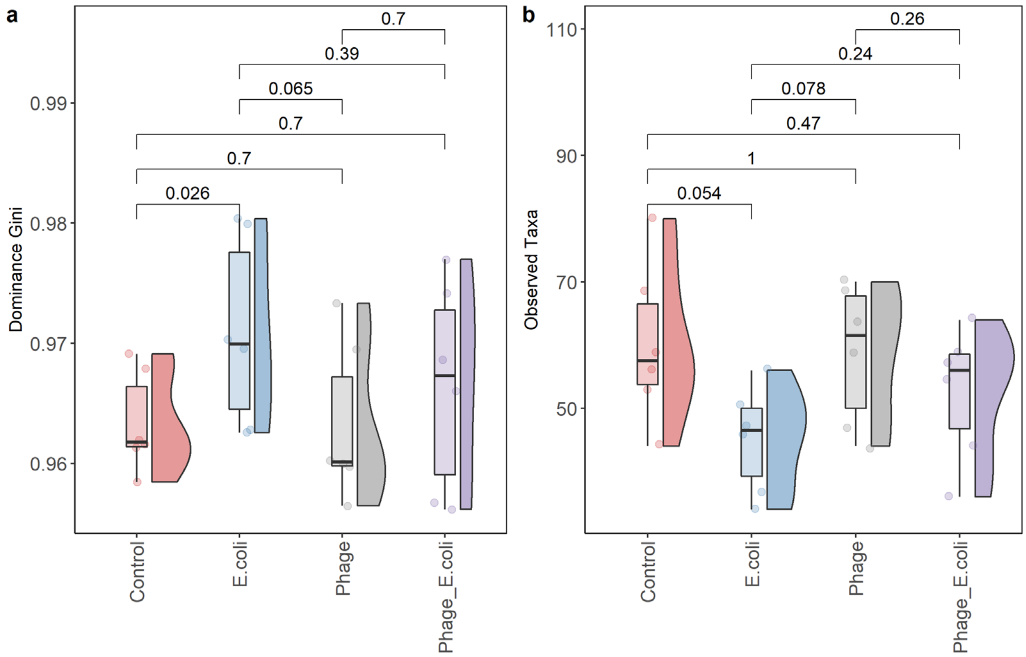

Supplementary Figure 2. Alpha diversity indices Dominance Gini (A) and Observed taxa (B) of faecal content at species level. Boxplot comparing the alpha diversity comparing all studied perturbations. Boxplots show that *E. coli* 5947 addition was the major perturbation for community composition abundance.

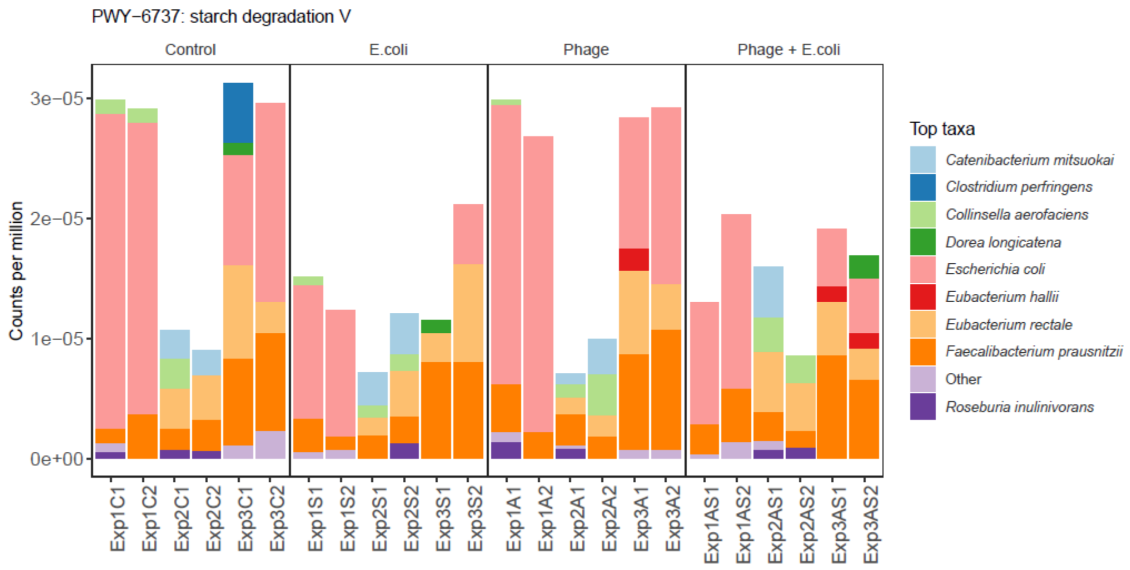

Supplementary Figure 3. Species contribution for Starch degradation V pathway (PWY:6739). Starch degradation associated pathways were detected using the HUMaN2 pipeline, and top contributing taxa for the starch degradation V pathway are represented (Notes: Exp1 – experiment using faecal content of donor one, Exp2 – experiment using faecal content of donor two, Exp3 – experiment using faecal content of donor three; C – control perturbation, S – *E. coli* 5947 addition, A – phage Ace addition, AS – *E. coli* 5947 and phage Ace addition in combination).

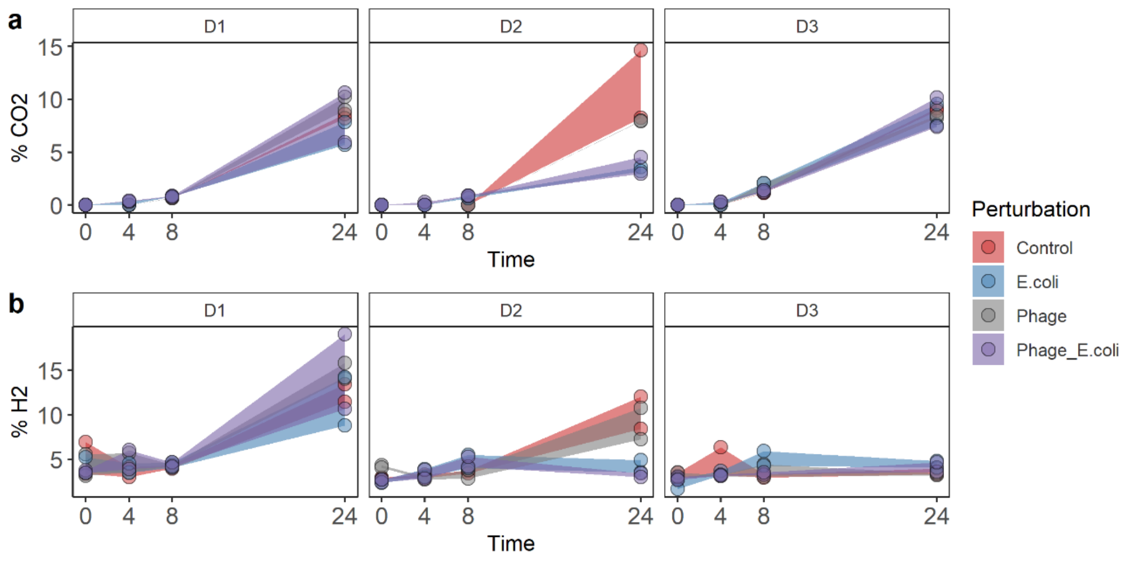

Supplementary Figure 4. Gas dynamics during fermentation. The monitoring of gas composition by GC during *in vitro* fermentation detected (A) carbon dioxide (CO_2) and (B) hydrogen (H_2). Different donors' faecal microbiota was used as inocula: donor 1 (D1), donor 2 (D2) and donor 3 (D3). The perturbations performed were as following: control - no perturbation, E.coli - only strain *E. coli* 5947 was added, Phage - only phage Ace was added, Phage_E.coli – phage Ace and *E. coli* 5947 were added in combination.

The monitoring of gas composition during fermentation showed the presence of hydrogen (H_2) and carbon dioxide (CO_2), during all time points, at different percentages for the different donors. The dynamics for both, H_2 and CO_2, were different in the presence of *E. coli* 5947, however, in the presence of phage Ace the dynamics were similar to the control condition.

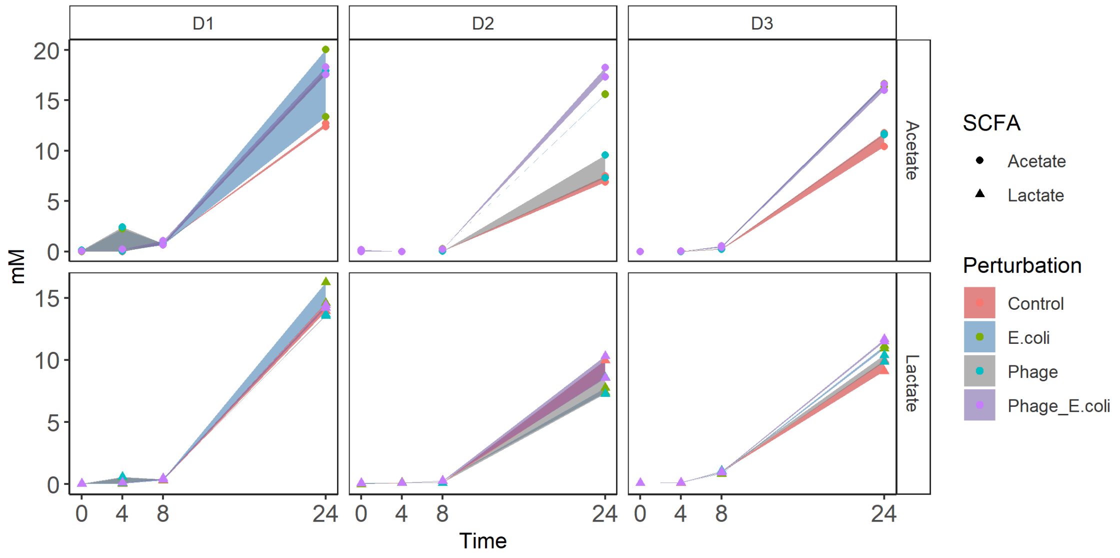

Supplementary Figure 5. Short chain fatty acid dynamics during fermentation. Non-gaseous metabolite production during in vitro fermentation was measured using HPLC. Acetate and lactate were detected. Different donors' faecal microbiota was used as inocula: donor 1 (D1), donor 2 (D2) and donor 3 (D3). The perturbation performed were as following: control - no perturbation, E.coli - only strain *E. coli* 5947 was added, Phage - only phage Ace was added, Phage_E.coli – phage Ace and *E. coli* 5947 were added in combination.

The same dynamics observed for gas productions were also observed for acetate during fermentation, which was more evident in D2 and D3 faecal fermentations. When adding *E. coli* 5947 (either alone or in the presence of phage Ace), an increase of acetate production was observed by the end of fermentation. Lactate was also detected by HPLC during fermentation, with increased concentrations by the end of all fermentations. No changes in lactate concentration were observed in the different perturbations (phage Ace and *E. coli* 5947) introduced.

Other SCFAs were also detected, however, the concentrations were low (below 5.0 mM), and for that reason they were not included in the final analysis.

Supplementary Methods

The simulated fluids stock solutions composition used in Methods section 2. Static *in vitro* digestion model is provided in Supplementary Table 1.

Supplementary Table 1. Preparation of stock solutions of simulated digestion fluids – Simulated Salivary Fuid (SSV), Simulated Gastric Fluid (SGF) and Simulated Intestinal Fluid (SIF). Volumes are calculated for a final volume of 500 mL, however it is recommended to make up the stock solution with distilled water to 400 mL (i.e. 1.25x concentrate) for storage at – 20 C. The volumes for SSF, SGF and SIF used in section 2 are referred to the 1.25x concentrate

			**SSF pH 7**		**SGF pH 3**		**SIF pH 7**	
**Constituent**	**Stock conc.**		**Vol. of stock**	**Final Conc.**	**Vol. of stock**	**Final Conc.**	**Vol. of stock**	**Final Conc.**
	$g\ L^{-1}$	$mol\ L^{-1}$	mL	$mmol\ L^{-1}$	mL	$mmol\ L^{-1}$	mL	$mmol\ L^{-1}$
KCl	37.3	0.5	15.1	15.1	6.9	6.9	6.8	6.8
KH_2PO_4	68	0.5	3.7	3.7	0.9	0.9	0.8	0.8
$NaHCO_3$	84	1	6.8	13.6	12.5	25	42.5	85
NaCl	117	2	-	-	11.8	47.2	9.6	38.4
$MgCl_2(H_2O)_6$	30.5	0.15	0.5	0.15	0.4	0.1	1.1	0.33
$(NH_4)_2CO_3$	48	0.5	0.06	0.06	0.5	0.5	-	
pH adjustment								
HCl		6	0.09	1.1	1.3	15.6	0.7	8.4
$CaCl_2(H_2O)_2$ *	44.1	0.3		1.5 (0.75)		0.15 (0.075)		0.6 (0.3)

* $CaCl_2(H_2O)_2$ is not added to the simulated digestion fluids, due to possible precipitation. Is added into the final mixture of simulated digestion fluid and food. In brackets is given the final Ca^{2+} concentration in the digestion mixture.
